# Psoromic Acid, a Lichen-Derived Molecule, Inhibits the Replication of HSV-1 and HSV-2, and Inactivates HSV-1 DNA Polymerase: Shedding Light on Antiherpetic Properties

**DOI:** 10.3390/molecules24162912

**Published:** 2019-08-11

**Authors:** Sherif T. S. Hassan, Miroslava Šudomová, Kateřina Berchová-Bímová, Karel Šmejkal, Javier Echeverría

**Affiliations:** 1 Department of Natural Drugs, Faculty of Pharmacy, University of Veterinary and Pharmaceutical Sciences Brno, Palackého tř. 1946/1, 612 42 Brno, Czech Republic; 2 Department of Applied Ecology, Faculty of Environmental Sciences, Czech University of Life Sciences Prague, Kamýcká 129, 165 21, Praha 6-Suchdol, Czech Republic; 3 Museum of Literature in Moravia, Klášter 1, 664 61 Rajhrad, Czech Republic; 4 Departamento de Ciencias del Ambiente, Facultad de Química y Biología, Universidad de Santiago de Chile, Casilla 40, Correo 33, Santiago 9170022, Chile

**Keywords:** antiherpetic, anti-enzymatic properties, lichen metabolites, HSV, HSV replication, psoromic acid

## Abstract

Psoromic acid (PA), a bioactive lichen-derived compound, was investigated for its inhibitory properties against herpes simplex virus type 1 (HSV-1) and type 2 (HSV-2), along with the inhibitory effect on HSV-1 DNA polymerase, which is a key enzyme that plays an essential role in HSV-1 replication cycle. PA was found to notably inhibit HSV-1 replication (50% inhibitory concentration (IC_50_): 1.9 μM; selectivity index (SI): 163.2) compared with the standard drug acyclovir (ACV) (IC_50_: 2.6 μM; SI: 119.2). The combination of PA with ACV has led to potent inhibitory activity against HSV-1 replication (IC_50_: 1.1 µM; SI: 281.8) compared with that of ACV. Moreover, PA displayed equivalent inhibitory action against HSV-2 replication (50% effective concentration (EC_50_): 2.7 μM; SI: 114.8) compared with that of ACV (EC_50_: 2.8 μM; SI: 110.7). The inhibition potency of PA in combination with ACV against HSV-2 replication was also detected (EC_50_: 1.8 µM; SI: 172.2). Further, PA was observed to effectively inhibit HSV-1 DNA polymerase (as a non-nucleoside inhibitor) with respect to dTTP incorporation in a competitive inhibition mode (half maximal inhibitory concentration (IC_50_): 0.7 μM; inhibition constant (*K*_i_): 0.3 μM) compared with reference drugs aphidicolin (IC_50_: 0.8 μM; *K*_i_: 0.4 μM) and ACV triphosphate (ACV-TP) (IC_50_: 0.9 μM; *K*_i_: 0.5 μM). It is noteworthy that the mechanism by which PA-induced anti-HSV-1 activity was related to its inhibitory action against HSV-1 DNA polymerase. Furthermore, the outcomes of in vitro experiments were authenticated using molecular docking analyses, as the molecular interactions of PA with the active sites of HSV-1 DNA polymerase and HSV-2 protease (an essential enzyme required for HSV-2 replication) were revealed. Since this is a first report on the above-mentioned properties, we can conclude that PA might be a future drug for the treatment of HSV infections as well as a promising lead molecule for further anti-HSV drug design.

## 1. Introduction

The infectious disease caused by herpes simplex virus has been known since ancient Greek times [1]. Both herpes simplex virus type 1 (HSV-1) and herpes simplex virus type 2 (HSV-2) are highly infectious and infect most of the world population for life by establishing latent infections from which they periodically reactivate [2,3]. HSV is a member of Herpesviridae, a large family of enveloped-DNA viruses that induce numerous clinically significant syndromes in both adults and neonates [4,5]. It has been declared that HSV-1 relates to oral or facial infection and encephalitis, whereas HSV-2 is commonly connected with genital infection. Moreover, HSV infection was reported to be associated with potential human immunodeficiency virus (HIV) infection and invasive cervical carcinoma [6,7]. In addition, HSV is recognized to be involved in many ocular diseases, including endotheliitis, neurotrophic keratopathy, and stromal keratitis. During the primary infection, HSV enters the nerve cells and later persists latently in sensory neurons and lesions at or near the point of entry into the host. Reactivation of latent HSV is frequently initiated during the deficiency of immunity and the virus can be subsequently transmitted to new hosts [1,8,9]. Herpesvirus DNA polymerases are key enzymes that play a central role during the viral replication cycle and hence recognized as important targets for the development of antiherpetic drugs [10,11,12].

Management of HSV infections continues to be one of the main targets for researchers and healthcare providers worldwide, where it cannot be controlled or managed by vaccination [13]. The standard treatment regimen for HSV relies on the use of acyclovir (ACV) and related nucleoside analogs that target HSV DNA polymerases. ACV-resistant HSV has become increasingly common, particularly among patients with HIV due to the extensive use of such medication as well as other related nucleoside analogs [14,15]. Natural products as important sources of biologically active molecules have been considered an alternative mean to conventional therapy, offering lower occurrence of resistance, various modes of action and reduced undesirable effects [1].

Psoromic acid (PA) is a natural molecule, which belongs to β-orcinol depsidones. This compound was detected in a wide range of lichen species and remarkably distributed in three genera—*Usnea*, *Psoroma*, and *Alectoria* [16]. Considering the diverse distribution of PA in lichens, this compound was considered to be a key standard for lichen chemotaxonomy [17]. Numerous studies have reported various biological properties of PA, such as antibacterial, antitumor, antigenotoxic, antiplasmodial, cytotoxic, antioxidant, cardiovascular protective effects, and anti-osteoporotic [18,19,20,21,22,23,24].

Recently, PA was found to possess antibacterial activity against *Mycobacterium tuberculosis* and to inhibit its associated enzymes, UDP-galactopyranose mutase and arylamine-*N*-acetyltransferase [17]. The present study aimed to investigate, for the first time, the antiviral properties of PA against HSV-1 and HSV-2 using in vitro replication assays. In addition, PA was subjected to a radiolabeled nucleotide-based assay to explore its anti-enzymatic activity against HSV-1 DNA polymerase and hence unveil the mechanism underlying its inhibitory effect on HSV-1 replication by targeting such an enzyme. Additionally, molecular docking analyses were conducted to further confirm the outcomes of in vitro results and to reveal the binding modes along with the molecular interactions of PA with the active sites of HSV-1 DNA polymerase and HSV-2 protease, which in turn authenticated the inactivation properties of PA towards both enzymes.

## 2. Results and Discussion

### 2.1. Antiviral Activity

#### Evaluation of Antiviral Activities against HSV-1 and HSV-2

The first point to be considered before performing antiherpetic assays is to determine the cytotoxicity of the test compound. Thus, we assessed the cytotoxic effect of PA, PA in combination with ACV, and standard ACV on Vero cells by the neutral red dye-uptake method. The results indicated that the CC_50_ values for the test compounds were greater than 310 µM (Table 1 and Table 2).

Furthermore, the effect of the test compounds on the replication of HSV-1 was assayed in infected Vero cells with HSV-1 using a plaque reduction assay. Based on the obtained results, PA exhibited greater anti-HSV-1 activity (50% inhibitory concentration (IC_50_): 1.9 μM; selectivity index (SI): 163.2) than ACV (IC_50_: 2.6 μM; SI: 119.2) (Table 1). It is noteworthy that PA with ACV in combination displayed potent antiviral activity against HSV-1 (IC_50_: 1.1 µM; SI: 281.8) compared with that of single ACV.

The antiviral effects against the replication of HSV-2 were evaluated by the titer reduction assay in Vero cells infected with HSV-2 using quantitative real-time reverse transcription PCR. PA presented similar anti-HSV-2 activity (50% effective concentration (EC_50_): 2.7 μM; SI: 114.8) compared with that of ACV (EC_50_: 2.8 μM; SI: 110.7) (Table 2). Interestingly, the potency of anti-HSV-2 activity was remarkably enhanced through the combination of PA with ACV (EC_50_: 1.8 µM; SI: 172.2) compared with that of ACV.

The selectivity index (SI; calculated as ratios CC_50_/IC_50_ and CC_50_/EC_50_) is vital to control any possible cytotoxicity of any molecule that might be confused with antiviral activity. Thus, the safety index of PA was validated by determining the SI, where PA revealed cytotoxicity against Vero cells (normal host cells) at a concentration higher than its IC_50_ and EC_50_.

In recent years, the evaluation of the combinatory outcome of standard antiviral medications in combination with compounds from different classes (as a combinatory treatment) is one of the most recommended strategies to overcome the resistance to antiviral drugs [25,26]. Thus, it is quite significant that the results obtained by combining PA with ACV have led to potent anti-HSV-1 and anti-HSV-2 properties associated with potential reduced resistance.

The last decade has witnessed a global concern with regards to the fast-growing rate of HSV infections worldwide. However, ACV and related synthetic nucleoside analogs are drugs of choice, and because they are widely available in the international markets for the treatment of HSV infections, their extensive use has led to the problem of drug-resistant strains [1,27]. As a result, treatment failure has developed. Thus, in order to overcome such hurdles, there is an urgent need to search for alternative sources that provide lower resistance, reduced undesirable effects, and various mechanisms of action [28]. Accordingly, in the current study, PA as a natural lichen metabolite shows a notable ability to inhibit the replication of HSV-1 and HSV-2 with an accepted degree of selectivity index.

### 2.2. Evaluation of Anti-HSV-1 DNA Polymerase Activity

Given the importance of PA in exhibiting a notable inhibitory potency against the replication of HSV-1, it is imperative to study its action against HSV-1 DNA polymerase, a primary enzyme required for the viral replication cycle. As presented in Table 3, PA revealed remarkable inactivation properties of the enzyme with respect to dTTP incorporation (IC_50_: 0.7 μM; *K*_i_: 0.3 μM) as compared with aphidicolin (IC_50_: 0.8 μM; *K*_i_: 0.4 μM) and ACV-TP with respect to dGTP incorporation (IC_50_: 0.9 μM; *K*_i_: 0.5 μM). Based on the obtained IC_50_ values, we applied a Cheng–Prusoff equation to calculate the absolute *K*_i_ values and ascertain the type of inhibition. The equation confirmed that PA, aphidicolin, and ACV-TP are competitive inhibitors of HSV-1 DNA polymerase with *K*_i_ values of 0.3, 0.4, and 0.5 μM, respectively. It has been reported that ACV competitively inactivates HSV DNA polymerases in a phosphorylated active form (ACV-TP). The inhibition process is initiated by converting ACV to ACV monophosphate by viral thymidine kinase, which is then converted by host cell kinases to the ACV-TP form [14]. Therefore, it is important to mention that PA competitively inhibited HSV-1 DNA polymerase (as a non-nucleoside inhibitor) directly without being activated to any active form; the same was observed with aphidicolin. It is known that drugs with pronounced inhibitory properties against HSV DNA polymerases can suppress the viral replication cycle [29,30]. Therefore, based on the obtained results, PA-induced HSV-1 DNA polymerase inhibition in a competitive inhibition fashion could principally contribute to its anti-HSV-1 activity.

Over the last few decades, several investigations and important review articles have declared synthetic or semi-synthetic non-nucleoside compounds with inactivation properties against herpesviruses DNA polymerase along with various mechanisms of action and specified modes of inhibition [31,32,33]. So far, limited studies only have reported natural-derived molecules as inhibitors of HSV DNA polymerases, such as oosporein and phosphonoacetic acid [34,35]. Unfortunately, these studies did not reveal or propose the type of inhibition induced. Thus, our study provided a deep investigation of the type of inhibition induced by PA against the target enzyme. Additionally, we report a first finding on the anti-HSV-1 DNA polymerase activity of PA.

### 2.3. Molecular Interactions of Psoromic Acid with HSV-1 DNA Polymerase

Based on our in vitro results and to better understand and clarify the mechanism underlying the antiherpetic effect of PA against HSV-1 via targeting HSV-1 DNA polymerase, we performed molecular docking analysis to reveal the binding mode and molecular interaction of PA with the active site of the enzyme.

**Figure 1:**
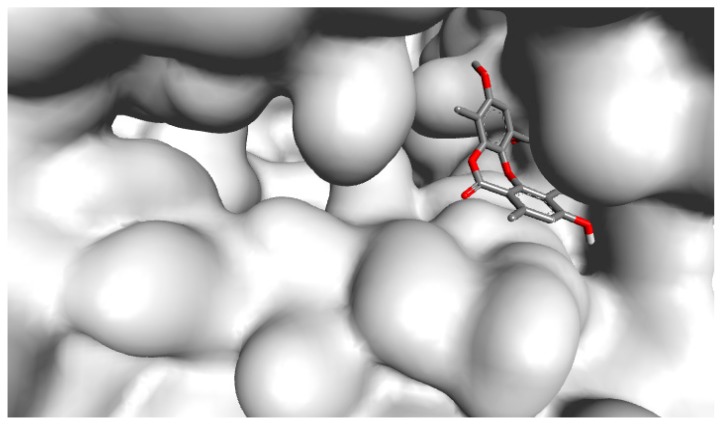
Molecular surface illustration of HSV-1 DNA polymerase, where psoromic acid efficiently binds to the active site of the enzyme.

HSV DNA polymerase plays a critical role during viral replication, and thus, drugs that target this protein can impair the viral replication cycle [36]. Our docking results revealed that PA was observed to bind remarkably to the active site of HSV-1 DNA polymerase and hence resulted in inhibiting the enzyme as displayed in a molecular surface model (Figure 1). The docking score for PA with HSV-1 DNA polymerase, which is expressed as binding affinity, was determined to be −8.2 kcal/mol.

**Figure 2:**
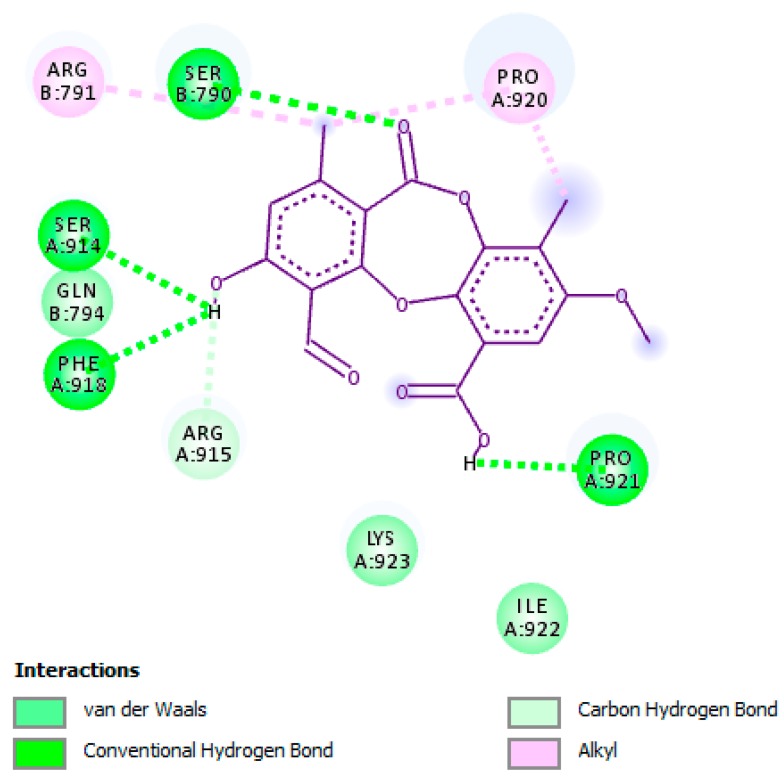
Molecular interaction of psoromic acid (PA) with the active site of HSV-1 DNA polymerase. Amino acid residues involved in HSV-1 DNA polymerase stabilization along with the hydrogen bonding and other essential interactions for enzyme inactivation are presented. The key functional groups of PA that are responsible for anti-HSV-1 DNA polymerase activity are depicted.

As shown in Figure 2, PA was observed to possess anti-HSV-1 DNA polymerase activity by forming hydrogen bonding interactions with residues Pro-A:921, Ser-A:914, Ser-B:790, and Phe-A:918, whereas with residue Arg-A:915 a carbon–hydrogen bonding interaction was formed. Moreover, several important hydrophobic and van der Waals interactions were detected. The hydroxyl, carbonyl, and methyl groups along with the phenyl ring of PA were declared as key functional groups responsible for the inhibitory activity against the enzyme by forming hydrogen bonding and hydrophobic interactions with important residues of the active site. Furthermore, all amino acid residues detected in the active site of HSV-1 DNA polymerase that accounted for the stabilization of the enzyme and formed the above-mentioned interactions with PA were previously described [37]. Since PA competes with the substrate and binds remarkably to the active site of the enzyme, and based on the in vitro anti-enzymatic results, PA is considered to be a competitive inhibitor.

### 2.4. Molecular Interactions of Psoromic Acid with HSV-2 Protease

Up to now, no 3D-crystal structure of HSV-2 DNA polymerase is registered in the RCSB Protein Data Bank. Thus, HSV-2 protease was selected as a drug target to further track the mechanism behind the inhibitory properties of PA towards HSV-2, and we carried out a molecular docking analysis to reveal this mechanism. Herpesvirus proteases have been recognized as belonging to a unique class of serine protease. These enzymes were found to be essential for viral replication and thus represent a feasible target for therapeutic intervention [38,39]. The serine residue of the active site (Ser-His-His catalytic triad) has been reported to be crucial for inactivating the herpesvirus protease [40]. Therefore, the inhibition of HSV-2 protease could be achieved by establishing classical interactions such as hydrogen bonding, hydrophobic and electrostatic between such residues in the active site of the enzyme and the inhibitor.

**Figure 3:**
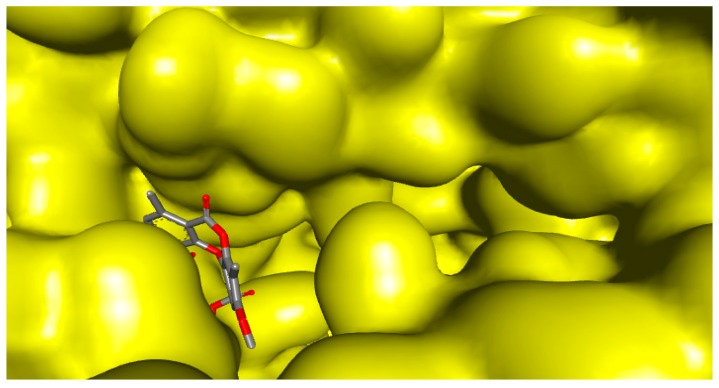
Molecular surface depiction of HSV-2 protease. As shown, psoromic acid binds remarkably to the active site of the enzyme.

**Figure 4:**
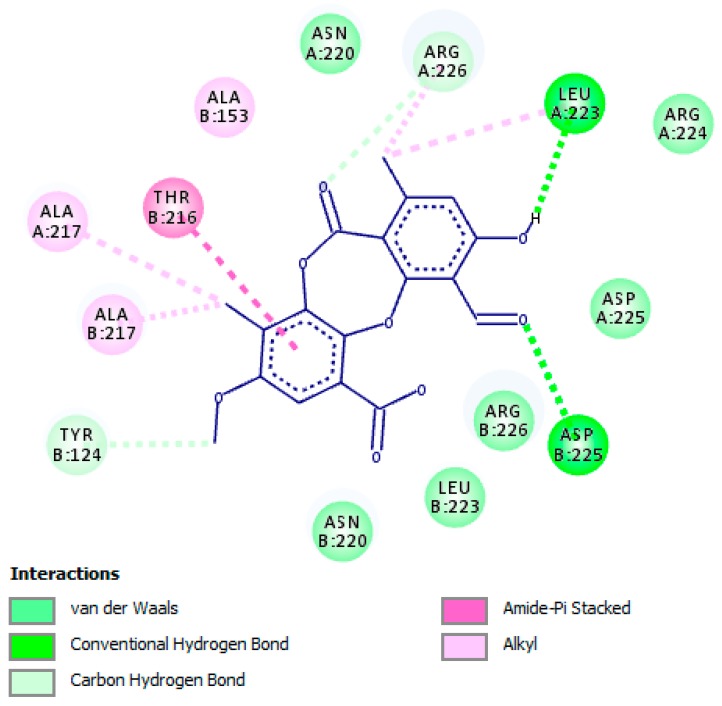
Molecular interaction of psoromic acid (PA) with the active site of HSV-2 protease. Amino acid residues involved in HSV-2 protease stabilization along with the hydrogen bonding and other essential interactions for enzyme inactivation are illustrated. Significant functional groups of PA that account for the inhibitory action against HSV-2 protease are presented.

The docking score for PA with HSV-2 protease, which is expressed as binding affinity, was determined to be −7.8 kcal/mol. The docking results confirmed that PA bound to the active site of HSV-2 protease (Figure 3) and hence inhibited the enzyme via establishing hydrogen bonding interaction between residue Leu-A:223 and the hydroxyl group of PA, whereas residue Asp-B:225 assigned hydrogen bonding interaction with the carbonyl group of PA (Figure 4). Carbon–hydrogen bonding interactions were also detected with residues Arg-A:226 and Tyr-B:124. Additional essential interactions were observed, including hydrophobic and van der Waals interactions that play a central role in the stabilization of PA as an inhibitor in the active site. Hydroxyl, carbonyl, and methyl groups, as well as the phenyl ring of PA, played a significant role in the inhibitory properties against the enzyme. Notably, all amino acid residues that were found close to the PA-HSV-2 protease binding site and established the above-mentioned interactions were previously reported to be essential for the enzyme stabilization [41,42]

It is important to mention that PA was not observed to bind to serine residue of the active site, which in turn indicates that PA bound to other catalytic residues of the active site. According to the results obtained from the docking investigation, PA was found to compete with the substrate and bound to the active site of the enzyme; therefore, we may suggest it as a competitive inhibitor.

## 3. Materials and Methods

### 3.1. Antiviral Activity

#### 3.1.1. Viral Strains, Medium, Cell Lines, and Reagents

Vero cells (ATCC: CCL 81™; London, UK), acquired from Motol University Hospital (MUH; Prague, Czech Republic) were seeded in Eagle’s minimum essential medium (MEM; Cultilab, Campinas, UK) supplemented with 10% fetal bovine serum (FBS; Gibco, Carlsbad, CA, USA), penicillin G (100 U/mL), streptomycin (100 μg/mL), and amphotericin B (25 μg/mL) (Sigma-Aldrich, Berlin, Germany), and maintained at 37 °C in 5% CO_2_.

Vero cells were infected with HSV-1 strain KOS (an ACV-sensitive strain) obtained from MUH, Prague, Czech Republic and propagated in Vero cells. Viral stocks were then titrated based on plaque forming unit (PFU) count by plaque assay as previously described [43]. Viral stocks were stored at −80^◦^C for further use.

A clinical isolate of HSV-2 (Strain-A234) (an ACV-sensitive strain; isolated from patients with HSV-2 genital infection) was kindly obtained from the MUH, Prague, Czech Republic. Quantitative real-time reverse transcription PCR was used to type the strain using primer pairs H_2_M_40_ 5′-GTACAGACCTTCGGAGG-3′ and H_2_P_4_ 5′-CGCTTCATCATGGGC-3′ for identification and further propagated in Vero cells. The titers which were expressed as 50% tissue culture infective dose (TCID_50_/mL) were determined by the cytopathic end-point assay as previously described [44]. Viral stocks were stored at −80 °C for further use.

#### 3.1.2. Determination of Cytotoxicity

The cytotoxic effect of standard PA (purity >98%; kindly obtained from University of Chemistry and Technology Prague, Prague, Czech Republic), PA in combination with ACV (Sigma-Aldrich, Berlin, Germany) as well as standard ACV on Vero cells was assessed using the neutral red dye-uptake assay as previously described with slight modification [44]. Briefly, the test compounds were dissolved in 0.2% dimethyl sulfoxide (DMSO). Further, stock solutions were prepared in deionized water and sterilized at a concentration of 620 μM. Vero cell monolayers cultivated in 96-well microtiter plates with two-fold serial dilutions of the test compounds were incubated for 48 h at 37°C in 5% CO_2_. After incubation, the morphological alterations of the treated cells were assessed using an inverted optical microscope (Leitz, Wetzlar, Germany) and the maximum non-toxic concentrations (MNTC) were determined. The concentrations of test substances that are necessary to decrease the cell viability by 50% (CC_50_) were assessed as compared with the untreated control cells. In order to acquire statistically relevant data for final evaluation, the cytotoxicity of test compounds was measured in at least three independent experiments conducted in duplicate.

#### 3.1.3. Anti-HSV-1 Assay

The inhibitory activity against the replication of HSV-1 was evaluated using a plaque reduction assay as previously described [43,45]. ACV was used as a reference drug. Briefly, cell monolayers were infected with 100 PFU of HSV-1 in MEM containing 1.5% carboxymethyl cellulose (CMC, Sigma-Aldrich, Berlin, Germany) in the presence or absence of test compounds at different concentrations. Afterward, the cells were subjected to incubation for 72 h at 37 °C, then fixed and stained using naphthol blue-black (Sigma-Aldrich, Berlin, Germany). Subsequently, the plaques were counted. IC_50_ values (concentrations of test compounds required to decrease the number of plaques by 50%) were determined as compared with untreated control cells. A selectivity index (SI) value was calculated as the ratio CC_50_/IC_50_.

#### 3.1.4. Anti-HSV-2 Assay

The titer reduction assay was used to evaluate the antiviral activity against the replication of HSV-2 following the previously reported assay [44,45]. ACV was used as a reference drug. Briefly, Vero cell monolayers were treated with test compounds at concentrations for which no changes were detected in cell morphology, and 80% of cell viability was ascertained. A 100 TCID_50_/mL portion of HSV-2 ACV-sensitive suspension was supplied to the treated and untreated cell cultures, which were then incubated at 37 °C for 48 h in 5% CO_2_. After incubation, the virus titers in the treated and untreated cells were determined. The antiherpetic activity was assessed as percentage inhibition (PI) using antilogarithmic TCID_50_ values as follows: PI = [1 − (antilogarithmic test value/antilogarithmic control value)] × 100. The dose–response curve was determined from the MNTC, and the 50% effective concentration (EC_50_) was assessed as the concentration essential for 50% protection against virus-induced cytopathic effects. SI value was calculated as the ratio CC_50_/EC_50_.

### 3.2. Inhibition of HSV-1 DNA Polymerase

#### 3.2.1. Preparation of HSV-1 DNA Polymerase

HSV-1 DNA polymerase was extracted from HSV-1-infected Vero cells following the procedure as previously designated [46]. Briefly, Vero cells were infected with HSV-1 (KOS strain) at a multiplicity of infection (MOI) equal to 5.0 for 12 h. Afterward, the infected cells were lysed with a buffer containing 0.25 M potassium phosphate (pH = 7.5), 10 mM 2-mercaptoethanol (2-ME), 1mM EDTA, 0.5% Triton X-100, 0.5 mM phenylmethane sulfonylfluoride (PMSF), and 20% glycerol, and subsequently sonicated and centrifuged at 10,000× *g* for 10 min at 4 °C. The resulting supernatant was additionally centrifuged at 100,000× *g* for 90 min at 4 °C and the supernatant was dialyzed against 10 mM potassium phosphate (pH = 7.5) with 10 mM 2-ME, 1mM EDTA, and 20% glycerol. Then, the obtained material was eluted in two ion-exchange chromatography columns (DEAE-cellulose and phosphate cellulose) (Sigma-Aldrich, Prague, Czech Republic) supplemented with potassium phosphate (0.02–0.5 mM) as previously described [47].

#### 3.2.2. Purification of HSV-1 DNA Polymerase

Extracted HSV-1 DNA polymerase was evaluated for its purity and possible contamination with cellular enzymes such as type B DNA polymerase (α and δ polymerases) using polyclonal antibodies raised against these cellular enzymes following the procedure as previously described [48]. The confirmation of the purity of HSV-1 DNA polymerase was achieved by using a kinetic assay in the presence or absence of ammonium sulfate (100 mM; an inhibitor of possible contaminant cellular polymerases) following the previously reported protocol [48].

#### 3.2.3. Anti-HSV-1 DNA Polymerase Activity

A radiolabeled nucleotide-based assay was used to evaluate the inhibitory properties of PA along with the standard drugs aphidicolin (a known competitive inhibitor of HSV-1 DNA polymerase with respect to dTTP incorporation; purchased from Sigma-Aldrich, Prague, Czech Republic with purity ≥98%) and ACV triphosphate (ACV-TP) (a known competitive inhibitor of HSV-1 DNA polymerase with respect to dGTP incorporation; obtained from Sierra Bioresearch (Tucson, AZ, USA) with purity >98%) against HSV-1 DNA polymerase based on the incorporation of dTTP as a substrate with some modifications [46,47]. Briefly, the enzyme assay was performed following the first-order kinetics conditions, where the enzymatic reaction initiated by pre-incubating 3 U/mL of HSV-1 DNA polymerase with a mixture containing 50 mM Tris-HCl (pH = 8), MgCl_2_ (8 mM), DTT (0.5 mM), bovine serum albumin (0.5 µg/mL), ammonium sulfate (100 mM; an inhibitor of possible contaminant cellular polymerases), 100 µM of each nucleotide (dATP, dGTP, dCTP, and [^3^H]-dTTP—0.5 µCi/nmol) (obtained from MUH, Prague, Czech Republic) and activated salmon-sperm DNA (12 µg/mL). Under standard assay conditions at 37 °C, 1U of HSV-1 DNA polymerase was found to incorporate 1 pmol of dTTP/min or 1 pmol of dGTP/min. [^3^H]-dGTP—0.5 µCi/nmol (obtained from MUH, Prague, Czech Republic) was used when the enzymatic assay was performed with ACV-TP.

The obtained mixture was further incubated with the test compounds (at concentrations ranging from 0.1 to 3 µM) at 37 °C for 30 min and afterward, the reaction was stopped by adding trichloroacetic acid (TCA; 10%). The resulting radioactivity was adsorbed over GF/C fiberglass membranes (Whatman; Sigma-Aldrich, Berlin, Germany) and counted by liquid scintillation (Tri-Carb 2600, Packard Inc., Downers Grove, IL, USA). All measurements were conducted in triplicate and IC_50_ values were calculated by nonlinear regression. K_i_ values (inhibition constant; concentration required to produce half maximum inhibition) were calculated using the Cheng–Prusoff equation as previously described [49].

### 3.3. Molecular Docking Studies

For the preparation of protein and ligand and the processing of docking analyses, the 3D-crystal structure of HSV-1 DNA polymerase (PDB ID: 2GV9) and HSV-2 protease (PDB ID: 1AT3) were downloaded from the RCSB Protein Data Bank, whereas the 3D-structure of PA (SDF file CID: 23725) was acquired from the PubChem database. The PyRx docking tool associated with Autodock VINA software (version 0.8, The Scripps Research Institute, La Jolla, CA, USA) was used to assess the binding modes of PA in the active sites of HSV-1 DNA polymerase and HSV-2 protease. All docking parameters, settings, calculations, protonation conditions, and overall charges were tracked as previously reported [17,50]. Discovery Studio Visualizer v19.1.0.18287 (BIOVIA, San Diego, CA, USA) was used to visualize the results and elucidate the intermolecular interactions with the active sites of both enzymes [51].

## 4. Conclusions

There is a growing interest in naturally derived products providing outstanding health merits with relatively safe profiles, useful for the treatment of various diseases including, but not limited to, HSV infections. The present findings indicated that PA exerted remarkable antiherpetic activities against HSV-1 and HSV-2 (refer to the Results and Discussion section). PA was observed to possess a low cytotoxic effect on normal host cells (Vero cells) at a concentration (CC_50_; expressed in micromolar scale) higher than concentrations (IC_50_ and EC_50_; expressed in micromolar scale) observed with inhibiting the replication of HSV-1 and HSV-2. Thus, the calculated SI of PA has confirmed its safety. It is important to highlight that the effectiveness of antiviral properties against HSV-1 and HSV-2 was enhanced via the combination of PA with ACV (as a combinatory treatment). Furthermore, PA as a non-nucleoside drug was found to competitively inhibit HSV-1 DNA polymerase (refer to the Results and Discussion section). This indicates that the mechanism by which PA exhibited inhibitory action towards the replication of HSV-1 is attributed to the inhibition of HSV-1 DNA polymerase. Moreover, molecular docking investigations confirmed the inhibitory properties of PA against HSV-1 DNA polymerase and HSV-2 protease through the remarkable binding affinity with the active sites of both enzymes, along with the formation of essential hydrogen bonding and hydrophobic interactions (refer to the Results and Discussion section). Eventually, additional investigations must be performed to reduce the possible adverse actions of PA by using amended delivery systems prior to its possible practical application. On the other hand, there is a demand for additional studies to be conducted on the inhibitory action of PA against HSV-2 DNA polymerase along with the determination of the type of inhibition. Additionally, further research focused on the authentication of its activity along with pharmacokinetic and pharmacodynamic properties in vivo and in clinical trials is required.

## Figures and Tables

**Table 1:** Antiviral activity against the replication of herpes simplex virus type 1 (HSV-1) and cytotoxicity properties.

Compound	CC_50_ (μM)	IC_50_ (μM)	SI (CC_50_/IC_50_)
PA	>310	1.9 ± 0.42	>163.2
PA combined with ACV	>310	1.1 ± 0.41	>281.8
ACV	>310	2.6 ± 0.38	>119.2

PRISM software version 8.0 (GraphPad Software, Inc., La Jolla, CA, USA was used for statistical analysis and calculations. Values presented are means ± standard errors (SE) of three to five independent measurements conducted in duplicate. Nonlinear regressions of concentration–response curves were used to determine CC_50_ and IC_50_ values. Anova followed by post-hoc comparison tests (Dunnett and Student-Newman-Kuels) were used to assess the differences between treatments with test compounds and positive control. Statistical significance was *p* < 0.05. SI, selectivity index calculated as the ratio CC_50_/IC_50_; PA, psoromic acid; ACV, acyclovir; CC_50_, 50% cytotoxic concentration; IC_50_, 50% inhibitory concentration.

**Table 2:** Antiviral activity against the replication of herpes simplex virus type 2 (HSV-2) and cytotoxicity properties.

Compound	CC_50_ (μM)	EC_50_ (μM)	SI (CC_50_/EC_50_)
PA	>310	2.7 ± 0.43	>114.8
PA combined with ACV	>310	1.8 ± 0.44	>172.2
ACV	>310	2.8 ± 0.32	>110.7

PRISM software version 8.0 (GraphPad Software, Inc., La Jolla, CA, USA) was utilized for statistical analysis and calculations. The presented values are means ± standard errors (SE) of three to five independent experiments performed in duplicate. Anova followed by post-hoc comparison tests (Dunnett and Student-Newman-Kuels) were used to assess the differences between treatments with test compounds and positive control. Statistical significance was *p* < 0.05. SI, selectivity index calculated as the ratio CC_50_/EC_50_; PA, psoromic acid; ACV, acyclovir; CC_50_, 50% cytotoxic concentration; EC_50_, 50% effective concentration.

**Table 3:** Inhibitory activity of psoromic acid (PA), aphidicolin, and acyclovir triphosphate (ACV-TP) against HSV-1 DNA polymerase.

Compound	IC_50_ (μM)	*K*_i_ (μM)
PA	0.7 ± 0.51	0.3 ± 0.42
Aphidicolin	0.8 ± 0.61	0.4 ± 0.54
ACV-TP	0.9 ± 0.63	0.5 ± 0.34

PRISM software version 8.0 (GraphPad Software, Inc., La Jolla, CA, USA) was used for statistical analysis. Values displayed are means ± standard errors (SE) of five independent measurements performed in triplicate. IC_50_, half maximal inhibitory concentration; *K*_i_, inhibition constant (concentration required to produce half maximum inhibition); PA, psoromic acid; ACV-TP, acyclovir triphosphate.
